# Human astrocytes secrete IL-6 to promote glioma migration and invasion through upregulation of cytomembrane MMP14

**DOI:** 10.18632/oncotarget.11515

**Published:** 2016-08-23

**Authors:** Weiliang Chen, Tongliang Xia, Donghai Wang, Bin Huang, Peng Zhao, Jian Wang, Xun Qu, Xingang Li

**Affiliations:** ^1^ Department of Otolaryngology, Qilu Hospital, Shandong University, Jinan, China; ^2^ Department of Neurosurgery, Qilu Hospital of Shandong University and Brain Science Research Institute, Shandong University, Jinan, China; ^3^ Key Laboratory of Otolaryngology, Chinese Ministry of Health, Jinan, China; ^4^ Department of Biomedicine, University of Bergen, Bergen, Norway; ^5^ Institute of Basic Medical Sciences, Qilu Hospital of Shandong University, Jinan, China

**Keywords:** astrocytes, glioma, invasion, IL-6, MMP14

## Abstract

The brain microenvironment has emerged as an important component in malignant progression of human glioma. However, astrocytes, the most abundant glial cells in the glioma microenvironment, have as yet a poorly defined role in the development of this disease, particularly with regard to invasion. Here, we co-cultured human astrocytes with human glioma cell lines, U251 and A172, in an in vitro transwell system in order to ascertain their influence on migration and invasion of gliomas. mRNA and protein expression assays were subsequently used to identify candidate proteins mediating this activity. Astrocytes significantly increased migration and invasion of both U251 and A172 cells in migration and invasion (plus matrigel) assays. Membrane type 1 matrix metalloproteinase (MMP14) originating from glioma cells was identified in qRT-PCR as the most highly up-regulated member of the MMP family of genes (~ 3 fold, *p* < 0.05) in this system. A cytokine array and ELISA were used to identify interleukin-6 (IL-6) as a highly increased factor in media collected from astrocytes, especially under co-culture conditions. IL-6 was also the key cytokine inducing cytomembrane MMP14 expression, the active form of MMP14, in glioma cells. Knockdown of MMP14 with siRNA led to decreased migration and invasion. Taken together, our results indicated that cytomembrane MMP14 was induced by IL-6 secreted from astrocytes, thereby enhancing the migration and invasion of glioma cells through activation of MMP2. Therefore, this IL-6 and MMP14 axis between astrocytes and glioma cells may become a potential target for treatment of glioma patients.

## INTRODUCTION

Glioblastoma multiforme (GBM) is the most common and devastating primary malignant tumor in the brain. Although surgery combined with radiotherapy plus chemotherapy improve survival, the prognosis remains poor with high recurrence rates and a median survival of only 14.6 months [1]. The invasive phenotype of GBM that makes tumor impossible complete resection as well as the resistance to the current therapeutic intervention is the major cause of poor prognosis in glioma patients [2]. Therefore, understanding the mechanisms that regulate glioma invasion and the development of novel strategies to inhibit this property are critical to improve poor prognosis for patients.

Recently, a number of studies have demonstrated that the tumor microenvironment, particularly the tumor stromal cells, contribute to the malignant behavior of human gliomas [3, 4]. Astrocytes are the most abundant glial cell in the human brain, comprising 50% of the brain volume [5]. They are a unique group of stromal cells in the glioma microenvironment, and in fact, the first cell type in the brain to surround and react to evolving tumor cells [6]. Studies have revealed that astrocytes enhance the invasion potential of glioblastoma stem-like cells [7] and play an important role in glioma growth, chemoresistance and angiogenesis [3, 8]. Additionally, astrocytes in the glioma microenvironment became functionally reactive [3] and have been shown to secrete a variety of cytokines, such as glia-derived neurotrophic factor (GDNF) and transforming growth factor β (TGF-β) which regulate glioma proliferation and invasion [9, 10]. While other cell types in the glioma microenvironment, such as microglia, have a well-established role in the development of glioma [11, 12], the function of astrocytes in this environment and their association with tumor invasion have not been fully elucidated.

One way that astrocytes might contribute to the invasive capacity of human gliomas is by promoting activity of matrix metalloproteinases. Membrane type 1 matrix metalloproteinase (MT1-MMP; MMP14), a transmembrane proteinase of MMP family, is critical for degradation of the extracellular matrix, for example. MMP14 enhances tumor invasion and dissemination through cleavage of CD44, a cell surface glycoprotein [13], or activation of MMP-2 and MMP9 through a combination of TIMP-2 and MMP14 [14]. A growing body of evidence indicates that MMP14 is up-regulated in diverse aggressive tumor and stromal cells, and thus, MMP14 originating from microglia or glioma cells is emerging as a potential interventional target for glioma therapy [15]. Interleukin-6 (IL-6), one of the growth and survival cytokines involved in the modulation of immune and inflammatory responses, has been recently shown to promote invasion and migration of glioma cells [12, 16]. IL-6 has also been found to activate MMP2 and MMP9 which enhances the invasion capability of cancer cells [17, 18]. Since MMP14 is an activator of MMP2 and MMP9, a possible mechanism driving tumor invasion and migration is the activation of MMP2 and MMP9 through IL-6 induced MMP14.

In the current study, we developed a model system in vitro in order to begin to characterize the function of human astrocytes in glioma migration and invasion. Using a transwell co-culture system, we found that astrocytes were able to induce MMP14 activity in glioma cells, thereby enhancing migration and invasion. The relationship between astrocytes and glioma cells was further dissected through the identification of IL-6 from protein arrays as a critical cytokine mediating an increase in cytomembrane MMP14. These results highlighted an important pathway of communication between astrocytes and glioma cells, the IL-6 and MMP14 axis, and perhaps a possible target for treatment of human gliomas.

## RESULTS

### Human astrocytes promote the migration and invasion of glioma cells *in vitro*

**Figure 1:**
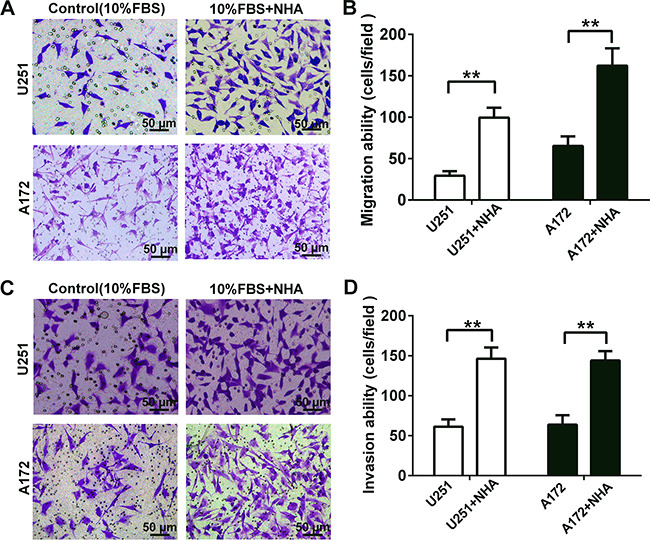
Human astrocytes significantly increase the migration and invasion of glioma cells by releasing soluble factors *in vitro* **A.** Representative micrographs of migratory cells in transwell migration assay containing U251 or A172 glioma cells (4 × 10^4^) in co-culture with astrocytes as indicated. NHA or DMEM containing 10% FBS (as the control) were added to the bottom chamber. Glioma cells suspended in serum-free DMEM were seeded in the upper chamber. After incubation for 24 h, cells which migrated through transwell membrane were fixed, stained and quantified. **B.** Graphic representation of the quantification of three independent experiments each using five random fields. **C.** Representative micrographs of invasive cells in transwell invasion assays (plus Matrigel). Glioma cells (8 × 10^4^) were seeded as indicated and co-cultured with NHA for 48 h. **D.** Graphic representation of the quantification of invasive cells. All data are shown as the mean ± SD of three independent experiments; ** *p* < 0.01; ***p < 0.001.

We developed a transwell co-culture model in order to establish whether astrocytes interact with glioma cells to promote migration and invasion. Invasion was distinguished from migration by the addition of matrigel to the upper chamber of the transwell system. Two glioma cell lines, U251 and A172, were evaluated. Migration and invasion of both U251 and A172 glioma cells in co-culture with astrocytes were significantly increased: U251, 2.4 and 1.3 fold, respectively; A172, 1.5 and 1.3 fold, respectively (Figure 1).

### Soluble factors secreted by astrocytes upregulate proteins and activate signaling pathways associated with migration and invasion

**Figure 2:**
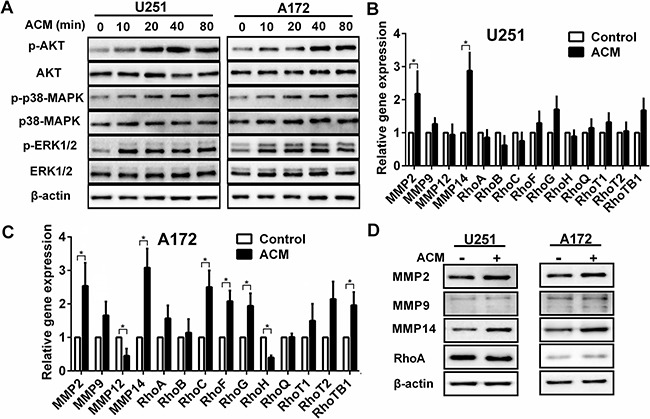
The activated signaling pathways and upregulation of gene and protein associated with invasion induced by astrocytes **A.** Western blot analysis of protein lysates prepared from U251 or A172 exposed to astrocytes condition medium (ACM) for the times indicated. **B, C.** Graphic representations of qRT-PCR results for invasion related gene expression changes induced in U251 or A172 in co-culture with astrocytes. Total RNA was extracted from U251 or A172 glioma cells were incubated with ACM for 48 h where DMEM containing 3% FBS was used as the control. * *p* < 0.05. **D.** Western blot analysis performed with protein lysates prepared U251 or A172 cells after incubation with ACM for 48 h. Proteins examined are indicated.

To explore the molecular mechanisms underlying the effect astrocytes might exert on glioma cells, activation of proteins and expression of genes were examined in signaling pathways known to have a role in migration and invasion. U251 and A172 cells were stimulated for 0, 10, 20, 40, and 80 min with culture media collected from astrocytes (ACM), and the phosphorylation of kinases in the AKT pathway was assessed by Western blot. In both cell lines, phosphorylation of AKT, p38MAPK and ERK1/2 was observed (Figure 2A). These results indicated that astrocytes were involved in promoting cancer migration and invasion [19, 20].

We also examined mRNA levels of MMP, Rho, ADAM and STAT family members that have been previously shown to be involved in glioma migration and invasion. Many genes, including MMP2, MMP9, RhoF, RhoG, RhoTB1, ADAM17, ADAM19, STAT3, STAT5 and STAT6, were up-regulated in both cell lines in response to ACM. However, MMP14 exhibited the most significant increase in expression in both cell lines (~ 3 fold, *p* < 0.05; Figure 2B and 2C, Figure S1A and S1B). Protein levels were correspondingly increased as observed on Western blot (Figure 2D). These results suggested that soluble factors secreted by astrocytes led to activation of the AKT, p38MAPK and ERK1/2 signaling pathway and up-regulated MMP14, thereby promoting migration and invasion of glioma cells.

### Astrocyte conditioned medium increases cytomembrane MMP14 expression

**Figure 3:**
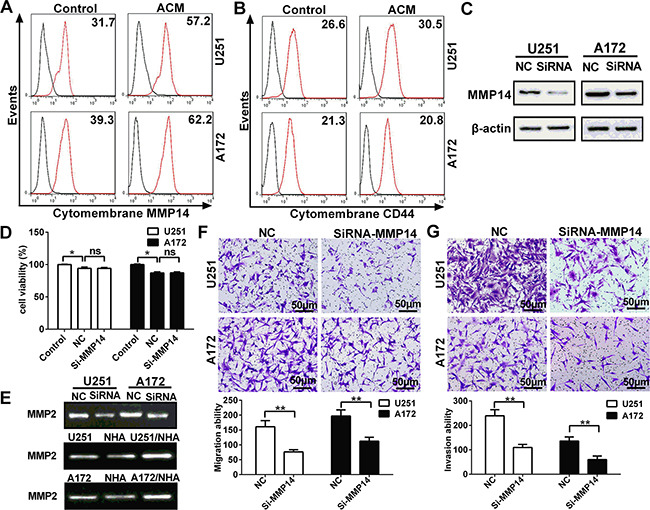
Cytomembrane MMP14 in glioma cell lines is up-regulated by astrocytes in glioma cell lines, and promotes invasion and migration through activation of MMP2 but not cleavage of CD44 **A.** Flow cytometry performed with anti-MMP14 and secondary antibody conjugated to Dylight 488 fluorescent dye to detect cytomembrane MMP14 expression on U251 or A172 cells after ACM stimulation for 48 h. **B.** Flow cytometry performed with FITC-anti-CD44 to determine levels of the cleavage of cytomembrane CD44 in glioma cells cultured with ACM for 48 h. **C.** Western blot analysis for MMP14 48 h after transfection of U251 or A172 cells with siRNA-MMP14 or negative control sequences (NC). **D.** Cell viability of U251 or A172 as determined by CCK-8 48 h after transfection with si-MMP14 or NC. **E.** Zymography gelatin gels showing activity of MMP2 as modulated by MMP14 expression or ACM. U251 or A172 cells transfected with siRNA-MMP14 or NC and analyzed for MMP2 activity after 48 h. MMP2 activity in U251 or A172 cells exposed to the conditioned medium indicated for 48 h. **F.** Transwell migration assay performed with U251 or A172 transfected with siRNA-MMP14 or NC (24 h) and co-cultured with astrocytes. After incubation for 24 h, cells which migrated through transwell membrane were fixed, stained and quantified. Representative micrographs and graphic representation of the quantification of three independent experiments each using five random fields. **G.** Transwell invasion assay (plus Matrigel) performed with U251 or A172 24 h after transfection with siRNA-MMP14 or NC. Representative micrographs are shown as well as graphic representation of the quantitation of the experiments. The data are shown as the mean ± SD in three independent experiments; *p < 0.05; **p < 0.01.

MMP14 is produced and secreted as an inactive zymogen in the cytoplasm, which is also known as pro-MMP. When pro-MMP reaches the cell surface, the catalytic site is exposed, which is essential for MMP activity, and thus renders the MMP active [21]. Therefore, using flow cytometry, we investigated whether expression of cytomembrane MMP14 in glioma cells was also increased upon exposure to ACM. Levels of cytomembrane MMP14 were increased by 80.4% and 58.3% on U251 and A172, respectively, after incubation with ACM for 48 h (Figure 3A). However, cytomembrane CD44, a substrate of MMP14, did not significantly change in parallel (Figure 3B, Figure S1E). The results that cleavage of cytomembrane CD44 was not coordinately increased along with MMP14 indicated that MMP14 might enhance glioma migration and invasion not through cleavage of CD44 but rather through activation of MMP family members.

To further establish a functional role for MMP14 in migration and invasion, siRNA was used to inhibit up-regulation while glioma cells exposure to ACM (Figure S1D). Results on Western blot revealed that up-regulated MMP14 protein was significantly reduced by the siRNA in both U251 and A172 cells (Figure 3C, Figure S1C). Importantly, si-MMP14 did not significantly affect viability of glioma cells relative to the negative control sequences (NC) group (Figure 3D). Transfection with siRNA was examined in functional assays for migration and invasion. First, gelatin zymography demonstrated that the increase in MMP14 induced by ACM in U251 and A172 also led to increased activity in MMP2 (Figure 3E). In contrast, MMP14 siRNA resulted in decreased activity of MMP2. Taken together, these results indicated that the activity of MMP2 was increased in glioma cells in co-culture (Figure 2B, 2C and 2D). Finally, knockdown of MMP14 resulted in decreased glioma invasion and migration (Figure 3F and 3G). These findings demonstrated that up-regulation of cytomembrane MMP14 by astrocytes promoted glioma migration and invasion through activation of MMP2 rather than cleavage of CD44.

### Cytomembrane MMP14 expression on glioma cells is upregulated by IL-6 secreted from astrocytes

**Figure 4:**
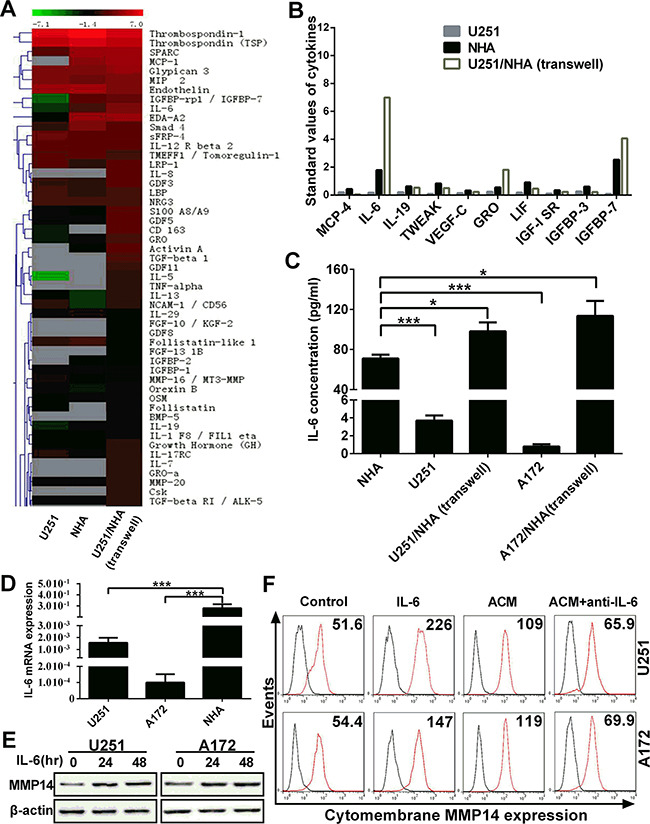
IL-6 secreted by astrocytes induces cytomembrane MMP14 expression on glioma cells **A.** Heat map and hierarchical clustering of cytokine array data. 507 cytokines were analyzed in the supernatant from monocultures of U251 and NHA, direct co-cultures of U251/NHA and transwell co-cultures of U251/NHA. The color scale shown at the top illustrates the relative expression level of an antibody in the certain slide: red represents high relative expression levels; green represents low relative expression levels. Gray represents a lower relative expression level of < 50 (the unreliable data). **B.** Up-regulated cytokines in NHA and co-cultures. Standard values of 11 cytokines which were significantly upregulated in NHA and co-cultures compared with U251 in monoculture. **C.** IL-6 concentrations in mono-culture and co-culture determined using ELISA. **D.** Graphic representation of the quantification of IL-6 mRNA performed with qRT-PCR on RNA isolated from the cells as indicated. **E.** Western blot analysis for total MMP14 protein performed with protein lysates prepared from U251 or A172 treated with IL-6 (50 ng/mL) for 48 h. **F.** Flow cytometry to detect levels of cytomembrane MMP14 in response to IL-6 or antibodies against IL-6 (anti-IL-6) in U251 or A172 cells exposed to ACM as indicated. **p < 0.01; ***p < 0.001.

The previous experiment demonstrated that soluble factors promoted glioma migration and invasion through up-regulation of cytomembrane MMP14. However, whether a specific factor was responsible for the up-regulation of MMP14 remained unknown. Therefore, we used arrays representing 507 cytokines to screen for candidate cytokines in the supernatant of media from U251, NHA and co-culture of NHA and U251 (Figure 4A, Figure S2). Ten cytokines were profoundly increased in media from NHA alone and U251/NHA co-culture, relative to U251 alone (Figure 4B). Of the 10 cytokines, IL-6 exhibited the greatest increase in NHA alone, and especially in the U251/NHA co-culture medium. This increase in IL-6 was confirmed in an ELISA for IL-6 and at the mRNA level with qRT-PCR (Figure 4C and 4D).

IL-6 has been reported to enhance tumor (including gliomas) migration and invasion through up-regulation or activation of MMP2 or MMP9 [16, 22, 23]. Glioma cells were therefore directly stimulated with IL-6 (50 ng/mL) to examine its role in MMP14 expression. Western blot analysis revealed an increase in the total protein for MMP14 in treated over untreated glioma cells (Figure 4E). In order to further confirm a role for IL-6 in MMP14 up-regulation, IL-6 antibodies were used to block function in glioma cells in culture. MMP14 protein levels were nearly completely abolished in glioma cells in the presence of IL-6 antibodies (Figure 4F). These results indicated that IL-6 was a key cytokine inducing cytomembrane MMP14 expression on glioma cells.

### Increased MMP14 and IL-6 expression levels are associated with advanced WHO grade and poor survival in patients

**Figure 5:**
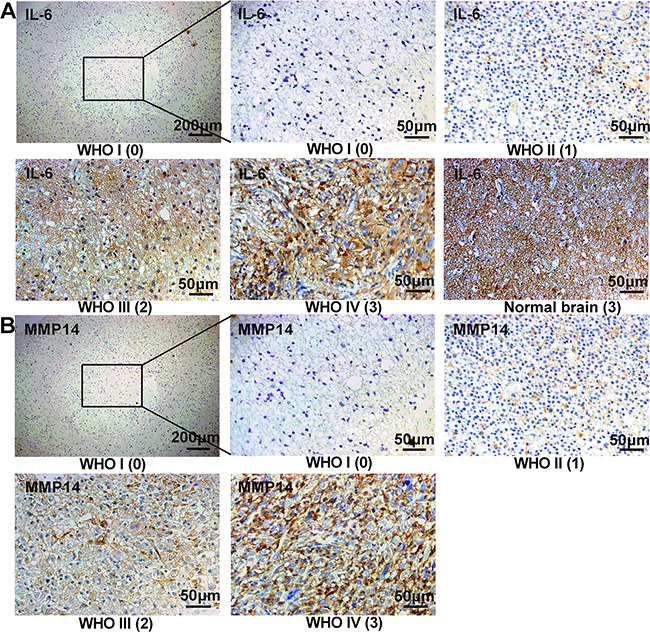
IL-6 and MMP14 are highly expressed in high grade gliomas from patients Immunohistochemistry and staining scores of IL-6 and MMP14 in different WHO grade glioma. Serial sections were used in individual cases for staining with each antibody. The number in brackets below the image is the immunohistochemistry staining score (0-3). Normal brain with a score of 3 had a high expression of IL-6 (A).

**Figure 6:**
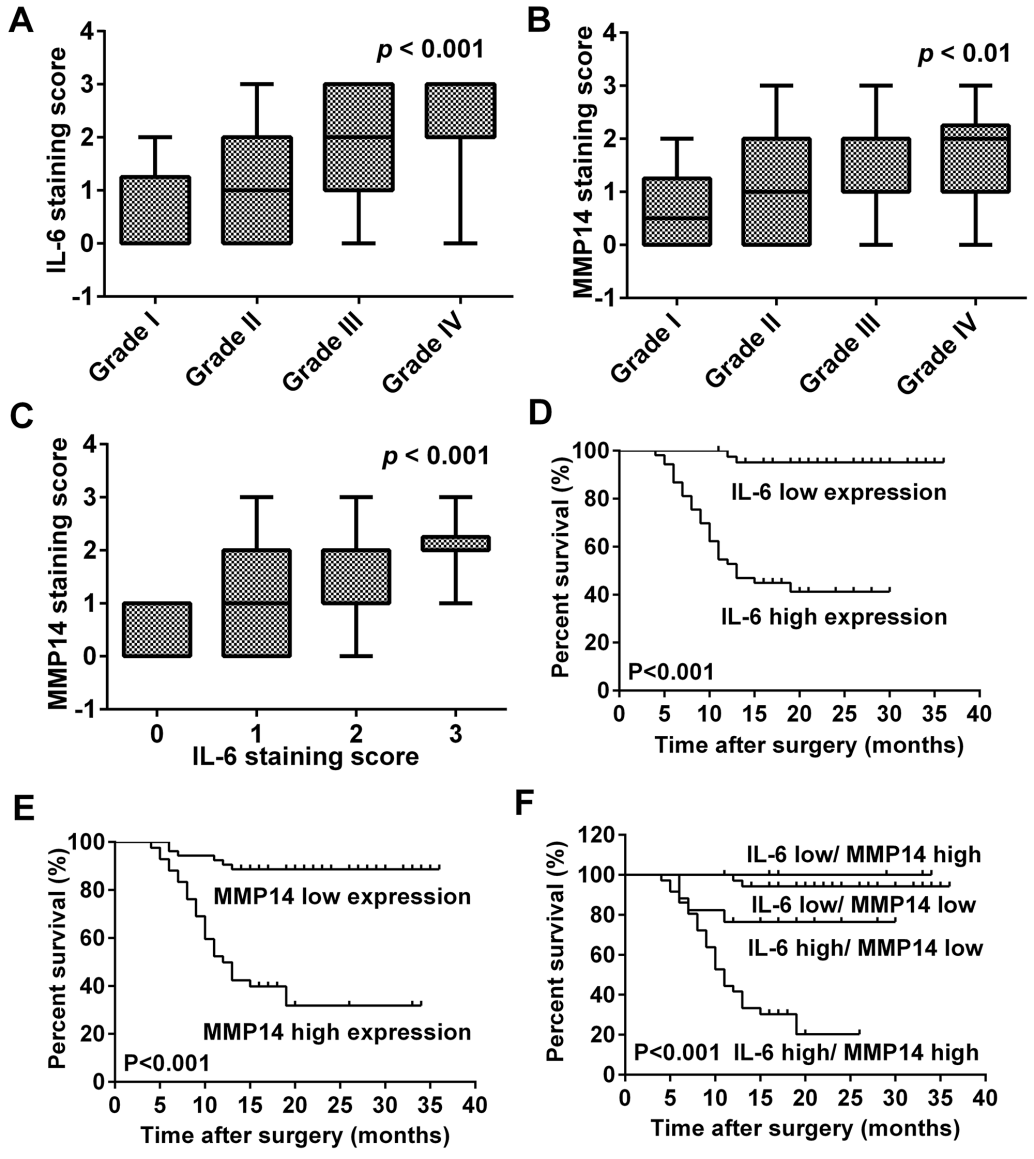
MMP14 expression is correlated with IL-6 in human glioma specimens and poor overall survival in glioma patients **A, B.** Correlation between IL-6 or MMP14 and glioma WHO grade. IL-6 and MMP14 expression were both correlated with glioma WHO grade (*p* < 0.001; *p* < 0.01). **C.** MMP14 expression was correlated with IL-6 expression (*p* < 0.001). Cumulative survival curves of glioma patients according to the respective protein expression levels of IL-6 and MMP14 protein based on immunohistochemistry scores. **D.** IL-6; **E.** MMP14; and **F.** IL-6 with MMP14. Both IL-6 (D) and MMP14 (E) expression correlated with poor overall survival on glioma patients. High co-expression of IL-6 and MMP14 was significantly associated with poor overall survival in patients (F, *p* < 0.001).

Immunohistochemistry was performed in order to examine IL-6 and MMP14 expression in human tumor samples (Figure 5) and to determine whether the proteins were associated with clinical parameters such as pathological grade and patient survival. We found that increased expression of MMP14 and IL-6 was associated with advanced WHO grade (Figure 6A and 6B, *p* < 0.001 and *p* < 0.01, respectively). More importantly, MMP14 expression was correlated with IL-6 (Figure 6C, *p* < 0.001). Normal brain with edema, also exhibited high expression of IL-6 (Figure 5A), demonstrated that IL-6 was expressed highly in astrocytes (confirming our hypothesis). Multivariate Cox regression analysis was used to evaluate the effect of multiple independent prognostic factors on survival (Table 1 and 2). All parameters tested including IL-6 and MMP14 expression were associated with poorer survival. Survival analysis as assessed by the Log-rank test demonstrated that expression of both IL-6 (Log-rank test, *p* < 0.001) and MMP14 (Log-rank test, *p* < 0.001) significantly correlated with poor overall survival (Figure 6D, 6E and 6F). These results demonstrated that MMP14 expression was correlated with IL-6 expression in patient samples and predicted poor overall survival in patients. The finding that MMP14 and IL-6 were correlated indicated that IL-6 might stimulate MMP14 expression as well.

**Table 1:** Clinical parameters of patients

Features	WHO I	WHO II	WHO III	WHO IV	*P* (survival)
Number of cases	10	29	22	34	
Age (mean years)	27.9	39.34	49.68	53.71	0.795
Sex					0.827
Male	3	19	12	20	
Female	7	10	10	14	
KPS score					0.493
≥ 80	10	27	17	23	
< 80	0	2	5	11	
Tumor size (largest tumor diameter)					0.007
≥ 5cm	2	6	6	18	
< 5cm	8	23	16	16	
Surgery					0.905
Gross total resection	10	29	21	33	
Partial resection	0	0	1	1	
Adjuvant treatment					0.005
Chemotherapy	0	0	3	7	
Radiotherapy	0	2	5	10	
Combination of chemotherapy and radiotherapy	0	0	1	2	
IL-6 expression (staining scores)					< 0.001
0	6	11	1	2	
1	2	9	6	5	
2	2	5	9	11	
3	0	4	6	16	
MMP14 expression (staining scores)					< 0.001
0	5	10	4	3	
1	3	11	8	9	
2	2	5	6	14	
3	0	3	4	8	

**Table 2:** Multivariate analysis of Cox proportional hazards model for multiple independent prognostic factors on overall survival

Parameter	Regression coefficient	Risk ratio	95% confidence interval	*P*
WHO grade	1.357	3.883	2.247-6.709	< 0.001
Tumor size	0.905	2.472	1.235-4.948	0.011
Adjuvant treatment	0.490	1.632	1.140-2.337	0.007
IL-6 expression	1.032	2.806	1.817-4.333	< 0.001
MMP14 expression	0.92	2.509	1.711-3.679	< 0.001

## DISCUSSION

Invasion is a complex process involving the interactions of cancer cells with stromal cells as well as the extracellular matrix [24]. As the most abundant stromal cell type in glioma microenvironment, human astrocytes have been found to promote proliferation and angiogenesis [3, 8], and invasion of glioma cells into the brain parenchyma, a critical factor contributing to patient mortality, has been shown to occur through direct contact between tumor cells and the stromal compartment [25, 26]. (Moreover, our previous work demonstrated that astrocytes could enhance chemoresistance in glioma through gap junctional communication [27].) However, few studies have investigated the functional role specifically of the astrocyte in invasion and migration in glioma. Here, we established a transwell co-culture model comprising human astrocytes and glioma cell lines, which provided a standardized and reproducible method for performing functional studies *in vitro*. The results indicated that astrocytes enhanced the migration and invasion potential of glioma, possibly through the secretion of IL-6 and increased MMP14 (Figure 1).

Autocrine secretion of cytokines has been shown to contribute to the infiltrative properties of glioma [28]. Our results indicate that cytokines originating from stromal cells in the tumor microenvironment may also promote invasive properties of human glioma cells. Previous studies have demonstrated that astrocytes modified gene expression in glioblastoma stem-like cells through the secretion of multiple factors and effectively enhanced invasion [7]. We found that soluble factors secreted from NHA induced glioma cells to express genes and proteins associated with migration and invasion (Figure 2B and 2C). Thus, our study corroborates previous findings. In addition, we found that AKT, p38MAPK and ERK1/2, signaling molecules associated with migration and invasion, were also activated in our model system [19, 20].

Interestingly, among the 14 genes examined from the MMP and Rho family, MMP14 was the most significantly up-regulated, with coordinate increase in protein levels as confirmed by Western blot. As a member of MMP family, MMP14 plays a vital role in the growth, migration, invasion and angiogenesis of tumor cells [29]. However, only upon reaching the cell surface is MMP14 activated and able to elicit catalytic activity [21]. MMP14 is more highly expressed in glioma than in normal cells, both *in vitro* and *in vivo*, and over-expression of MMP14 has been reported to enhance migration and invasion in glioma through several pathways [15]. MMP14 has been shown to increase pro-MMP2 and -MMP9 activity, mediate proteolysis of extracellular matrix and cleave CD44. We confirmed cytomembrane expression of MMP14 by flow cytometry in U251 and U172 cells. However, CD44 did not decrease in response to the increases in MMP14 induced by ACM, but siRNA knockdown of MMP14 did lead to decreased activity of MMP2 (Figure 3). These results suggested that MMP14 enhances glioma migration and invasion through the activation of MMP2 rather than through cleavage of CD44.

Cytokine arrays were performed with media from cell types in mono- and co-culture conditions in order to identify putative factors from astrocytes that might influence the expression of genes associated with migration and invasion in gliomas. This analysis revealed several cytokines that were associated not only with migration and invasion, but also with growth and angiogenesis in gliomas, such as MCP-4 [30], IL-6 [16], IL-19 [31], TWEAK [32], VEGF-C [33], GRO [34], LIF [35], IGF-1 SR, IGFBP-3 [36] and IGFBP7 [37, 38] (Figure 4A and 4B). Moreover, IGFBP-3 and IGFBP7 which are generally associated with inhibiting invasion were also found to be enriched in ACM. Given the number of potential migration and invasion related cytokines secreted by astrocytes, multiple paracrine cytokines are likely to act individually or in combination to regulate the migration and invasion of glioma cells.

Among these cytokines, IL-6 was found to be the most significantly increased factor in media from NHA, especially from NHA in co-culture with glioma cells (Figure 4). Recently, IL-6 has been found to promote tumor invasion and angiogenesis, in addition to proliferation and survival, but through upregulation or activation of MMP2 and MMP9 [16, 22]. We thus hypothesized that IL-6 was also involved in regulating MMP14 expression through the activation of MMP2 or MMP9 in glioma. Both total protein and the cytomembrane form of MMP14 were increased in glioma cells stimulated with IL-6. In support of a link between these two proteins, an IL-6 neutralizing antibody nearly completely abolished the up-regulation of MMP14 induced by ACM (Figure 4E and 4F). In addition, IL-6 activated the AKT, p38MAPK and ERK1/2 signaling pathway [39]. However, we did not determine whether other cytokines were also involved in the regulation of MMP14 expression.

We also investigated whether IL-6 and MMP14 protein levels might be associated with the clinical progression of the disease. Increased expression of IL-6 and MMP14 was first validated in primary human glioma specimens and then correlated with various clinical parameters. Both increased IL-6 and MMP14 expression correlated with advanced WHO grade and poor overall survival in glioma patients (Figure 5). Using analysis of relevance, we found that expression of MMP14 was associated with IL-6, which supported our hypothesis.

Although IL-6 has been traditionally considered to be secreted by glioma cells [40], the source of IL-6 in glioma specimens still remains a controversial issue. Our data however indicated that IL-6 was mainly (could be) secreted by infiltrating astrocytes. IL-6 thus stimulated glioma cells through a paracrine mechanism in our model system.

Taken together, our results demonstrated that migration and invasion of glioma cells was promoted through increased MMP14 expression regulated by the secretion of IL-6 from astrocytes. Furthermore, increased MMP14 expression correlated with IL-6 expression in primary human glioma specimens. Finally, expression levels were associated with advanced WHO grade and poor overall survival. Therefore, molecular characterization of cells within the tumor microenvironment, particularly the astrocyte, is important for the understanding and potentially the treatment of the invasive behavior of glioma. Blockade of the IL-6-MMP14 axis may thus provide a therapeutic strategy for the treatment of glioma and ultimately improve patient survival.

## MATERIALS AND METHODS

### Ethics statement

The study was approved by the Ethics Committees of Qilu Hospital of Shandong University (approval number: KYLL-2013-010) was performed in compliance with the Helsinki Declaration. Informed written consent was obtained from each individual.

### Patients and samples

Glioma specimens (*n* = 95) were obtained from patients during surgery at the Department of Neurosurgery, Qilu Hospital of Shandong University. Pathological examination of tumors and their classification were confirmed by an experienced pathologist in our hospital. A total of 55 males and 40 females (1.375:1) were enrolled in our study, and the median age was 48 yr. Pathological diagnoses were distributed among low and high grade tumors as follows: grade I, *n* =10; grade II, *n* = 29; grade III *n* = 22; and grade IV, *n* = 34). Clinical data for patients and tissue samples is in Table 1. None of patients received chemotherapy or radiotherapy before surgery, and KPS score was performed preoperatively. Normal brain tissue (*n* = 5) was obtained during surgical decompression of patients with hernia. The overall survival time was determined from the date of surgery until death or last follow-up.

### Cell lines, cell culture and antibodies

Normal human astrocytes (NHA) were purchased from Lonza (Walkersville, MD, USA), and human malignant glioma cell lines, U251 and A172, were obtained from the Chinese Academy of Sciences Cell Bank of Type Culture Collection (CBTCCCAS; Shanghai, China). NHA were cultured in Astrocytes Medium BulletKit^TM^ (Lonza) according to the manufacturer's instructions; the cells were not used beyond passage three and stained positive for the marker glial fibrillary acidic protein (GFAP). Glioma cells were cultured in Dulbecco's Modified Eagle Medium (DMEM; Hyclone, Logan, USA) with 10% fetal bovine serum (FBS). All cells were cultured at 37°C in a 5% CO_2_ humidified incubator. The following human antibodies were used: β-actin (Beyotime, Haimen, Jiangsu, China); MMP14, IL-6 (Abcam, Cambridge, UK); ERK1/2, p-ERK1/2, p38MAPK, p-p38MAPK, AKT, p-AKT, MMP2, MMP9, RhoA (Cell Signaling Technology, Boston, MA, USA), CD44-FITC (BD Biosciences, Heidelberg, Germany).

### Transwell migration and invasion assays

Assays were performed in 24-well transwell chambers (8.0 μm diameter pore). For the invasion assay, the upper chamber was coated with Matrigel (1:8 dilution; 80 μL; BD Bioscience). The lower chamber was seeded with the NHA, and after 24 h, 4×10^4^ or8×10^4^ glioma cells (migration and invasion, respectively) in serum-free DMEM (100 μL) glioma cells were added to the upper chamber. DMEM containing 10% FBS was used as the control. After co-culture for 24 or 48 h (migration and invasion, respectively), transwell membranes were fixed with methanol and stained with crystal violet. Glioma cells accumulating on the other side of the transwell membrane were quantified as the mean count of stained cells in six random fields under bright field microscopy (400× magnification).

### RNA isolation and quantitative real-time PCR

After incubation with ACM or transfection with siRNA for 48 h, total RNA was isolated from glioma cells using TRIzol. cDNA was synthesized with the ReverTra Ace qPCR RT Kit (Toyobo) according to the manufacturer's instructions. mRNA expression levels of different genes was determined with qRT-PCR. The target gene expression levels were normalized to GAPDH levels in the same reaction. Primer sequences were obtained from Primer Bank as follows (forward and reverse, respectively): MMP2, 5′-CAAGTTTCCATTC CGCTTC-3′ and 5′-GTTCCCACCAACAGTGGAC A-3′, MMP9, 5′-TTGACAGCGACAAGAAGTGGG-3′ and 5′-GCCATTCACGTCGTCCTTAT-3′, MMP14, 5′-CCCCGAAGCCTGGCTACA-3′ and 5′-GCATCA GCTTTGCCTGTTACT-3′, RhoA, 5′-GGAAAGCAGG TAGAGTTGGCT-3′ and 5′-GGCTGTCGATGGAAAA ACACAT-3′, RhoB, 5′-ATCCCCGAGAAGTGGGTC C-3′ and 5′-CGAGGTAGTCGTAGGCTTGGA-3′, RhoC, 5′-GGAGGTCTACGTCCCTACTGT-3′ and 5′-CGCAGTCGATCATAGTCTTCC-3′, RhoF, 5′-CCC CATCGGTGTTCGAGAAG-3′ and 5′-GGCCGTGTCG TAGAGGTTC-3′, RhoG, 5′-ACTAACGCTTTCCCCA AAGAG-3′ and 5′-GTGTACGGAGGCGGTCATAC-3′, RhoH, 5′-ATGCTGAGTTCCATCAAGTGC-3′ and 5′-T CTGCCTGCTGGTAGGACA-3′, RhoQ, 5′-CCACCGT CTTCGACCACTAC-3′ and 5′-AGGCTGGATTTACCA CCGAGA-3′, RhoT1, 5′-AAGGTAACAAGTCGATGG ATTCC-3′ and 5′-TCAGGTTTTTCGCTGAACACT-3′, RhoT2, 5′-GCATCCTGTTACTGGGCGAG-3′ and 5′-C GGCTTCTGAGTAGTCCACG-3′, RhoTB1, 5′-ATGGA CGCTGACATGGACTAC-3′ and 5′-ATCCCGAGAAC GCTCCAAGA-3′, IL-6, 5′-ACTCACCTCTTCAGAA CGAATTG-3′ and 5′-CCATCTTTGGAAGGTTCAGGT TG-3′, GAPDH, 5′-GGTGGTCTCCTCTGACTTCAAC AG-3′ and 5′-GTTGCTGTAGCCAAATTCGTTGTG-3′.

### Western blot

Glioma cells were treated with ACM for the time indicated or transfected with siRNA for 48 h. Protein lysates were prepared, separated by SDS-polyacrylamide gel electrophoresis and transferred to polyvinylidene difluoride membranes. Membranes were incubated with primary antibodies (1:1,000) overnight at 4°C and subsequently the appropriate secondary antibodies (1:5,000). Proteins were visualized using Western Chemiluminescence HRP substrate (Millipore Corporation, Billerica, MA, USA), and images were obtained and quantified with the Image Station 4000MM Pro (Carestream Health Inc., Woodbridge, MA, USA).

### Flow cytometry for the determination of MMP14 and CD44 cytomembrane expression

After incubation with ACM or IL-6 (50 ng/mL) for 48 h, glioma cells were collected and fixed in 4% paraformaldehyde. MMP14 cytomembrane expression was measured using an indirect flow cytometry protocol; cells were probed sequentially with MMP14 antibodies and secondary antibodies conjugated to Dylight 488 fluorescent dye, according to the manufacturer's instructions. CD44 expression was measured with direct flow cytometry and probed with CD44-FITC conjugated antibodies. Isotype IgG antibody was served as negative control.

### Zymography

Supernatants (serum-free DMEM) were collected from cultures which had been transfected with siRNA or co-cultured with NHA for 48 h. Supernatants (20 μL) mixed with 5× loading buffer (5 μL) was added for SDS-PAGE. Electrophoresis was run for 1.5 h at 100kv. The gels were washed 2 × 40 min in the first eluant (2.5% Triton X-100, 50mmol/L Tris –Hcl and 5mmol/L CaCl_2_), washed for 2× 40 min in the second eluant (containing 50mmol/L Tris –Hcl and 5mmol/L CaCl_2_) followed by incubation in the substrate buffer (pH 7.6) for 16 h at 37°C. Gels were stained with coomassie brilliant blue for 2 h, destained in acetic acid for 1 h, and imaged.

### Transfection of siRNA and cell viability assay

SiRNA targeting human MMP14 and non-targeted siRNA were purchased from GenePharma. Sequences of the siRNAs are as follows: 5′-AACAGGCAAAGCUGAUGCAGAdTdT-3′ (sense) and 5′-AAUCUGCAUCAGCUUUGCCUGdTdT-3′ (antisense) for MMP14 siRNA, 5′-UUCUCCGAACGUGUCACGUTT-3′ (sense) and 5′-ACGUGACACGUUCGGAGAATT-3′ (antisense) for non-targeted siRNA as a negative control. At 80% confluency, glioma cells were transfected with siRNAs (100 nM) for 6 h using Lipofectamine 2000 (Invitrogen) according to the manufacturer's instructions. Cell viability was measured with CCK-8 according to the manufacturer's instructions. Transfection efficiency was evaluated by Western blot 48 h later.

### Cytokine/Chemokine Array and IL-6 ELISA

A cytokine array was used to detect levels of cytokines or chemokines in supernatants from U251, NHA and transwell co-culture systems. U251 and NHA were seeded in 6-well plates at a density of 10×10^4^ cells per well or co-cultured in direct contact or in the transwell system. After incubation for 24 h, cultures were rinsed with DMEM, and media were replaced by DMEM containing 3% FBS. Supernatants were collected 48 h later and stored at −80°C. Samples were analyzed for the level of 507 cytokines using the RayBio® Biotin Label-based Human Antibody Array I, according to manufacturer's instructions (KangChen Bio-tech; Shanghai, China). IL-6 concentrations in these cultures were measured using a human IL-6 enzyme-linked immunosorbent assay (ELISA), according to the manufacturer's instructions.

### Immunohistochemistry

Formalin-fixed paraffin embedded human glioma specimen sections were deparaffinized, rehydrated, boiled in sodium citrate buffer (pH = 6.0) for antigen retrieval, incubated with 3% hydrogen peroxide in order to quench endogenous peroxidase activity and blocked with 10% goat serum to reduce nonspecific staining. Sections were incubated with primary antibodies against MMP14 (rabbit monoclonal, 1:100) or IL-6 (rabbit polyclonal, 1:400) overnight at 4°C and subsequently with poly-HRP secondary antibodies for 30 min. Sections were developed with diaminobenzidine and counterstained with hematoxylin. Images were captured using an Olympus IX81 microscope.

Immunohistochemistry staining was assessed by two independent pathologists. The number of positive staining cells in six representative fields was counted. Immunohistochemistry staining scores were defined by the percentage of positive cells as follows: 0 (0%), 1 (1-10%), 2 (11-50%) and 3 (> 50%) scores. Scores of 0 and 1 were classified as low expression, while scores of 2 and 3 were classified as high expression.

### Statistical analyses

Data was analyzed using SPSS software, version 20.0 (2011, IBM Corp.; Armonk, NY, USA). All experiments were repeated at least three times, and all data are presented as the mean ± SD. A statistical comparison between samples was determined by the independent-samples *t* test. The relationship between IL-6 or MMP14 and glioma WHO grade was evaluated using Spearman analysis, and survival curves were calculated using Kaplan-Meier analysis. The Log-rank test was used to assess differences in survival. The Cox proportional hazards model was used for multivariable analysis to evaluate the effect of multiple independent prognostic factors. Statistical significance is indicated as follows: * *p* < 0.05, ** *p* < 0.01, and *** *p* < 0.001.

## SUPPLEMENTARY FIGURES


