# Correction: Human astrocytes secrete IL-6 to promote glioma migration and invasion through upregulation of cytomembrane MMP14

**DOI:** 10.18632/oncotarget.28211

**Published:** 2022-03-03

**Authors:** Weiliang Chen, Tongliang Xia, Donghai Wang, Bin Huang, Peng Zhao, Jian Wang, Xun Qu, Xingang Li

**Affiliations:** ^1^Department of Otolaryngology, Qilu Hospital, Shandong University, Jinan, China; ^2^Department of Neurosurgery, Qilu Hospital of Shandong University and Brain Science Research Institute, Shandong University, Jinan, China; ^3^Key Laboratory of Otolaryngology, Chinese Ministry of Health, Jinan, China; ^4^Department of Biomedicine, University of Bergen, Bergen, Norway; ^5^Institute of Basic Medical Sciences, Qilu Hospital of Shandong University, Jinan, China; ^*^These authors have contributed equally to the work

**This article has been corrected:** In Figure 2A, the western blot image of p-p38-MAPK in the ‘U251’ cell line is an accidental duplicate of the image of p-p38-MAPK in the ‘A172’ cell line. The corrected Figure 2 is shown below. In addition, to address concerns regarding the contrast of the western blot images in Figures 3C and 4E, the original unprocessed images for these figures are shown below. All updated figures were produced from the original data. The authors declare that these corrections do not change the results or conclusions of this paper.

Original article: Oncotarget. 2016; 7:62425–62438. 62425-62438. https://doi.org/10.18632/oncotarget.11515

**Figure 2:**
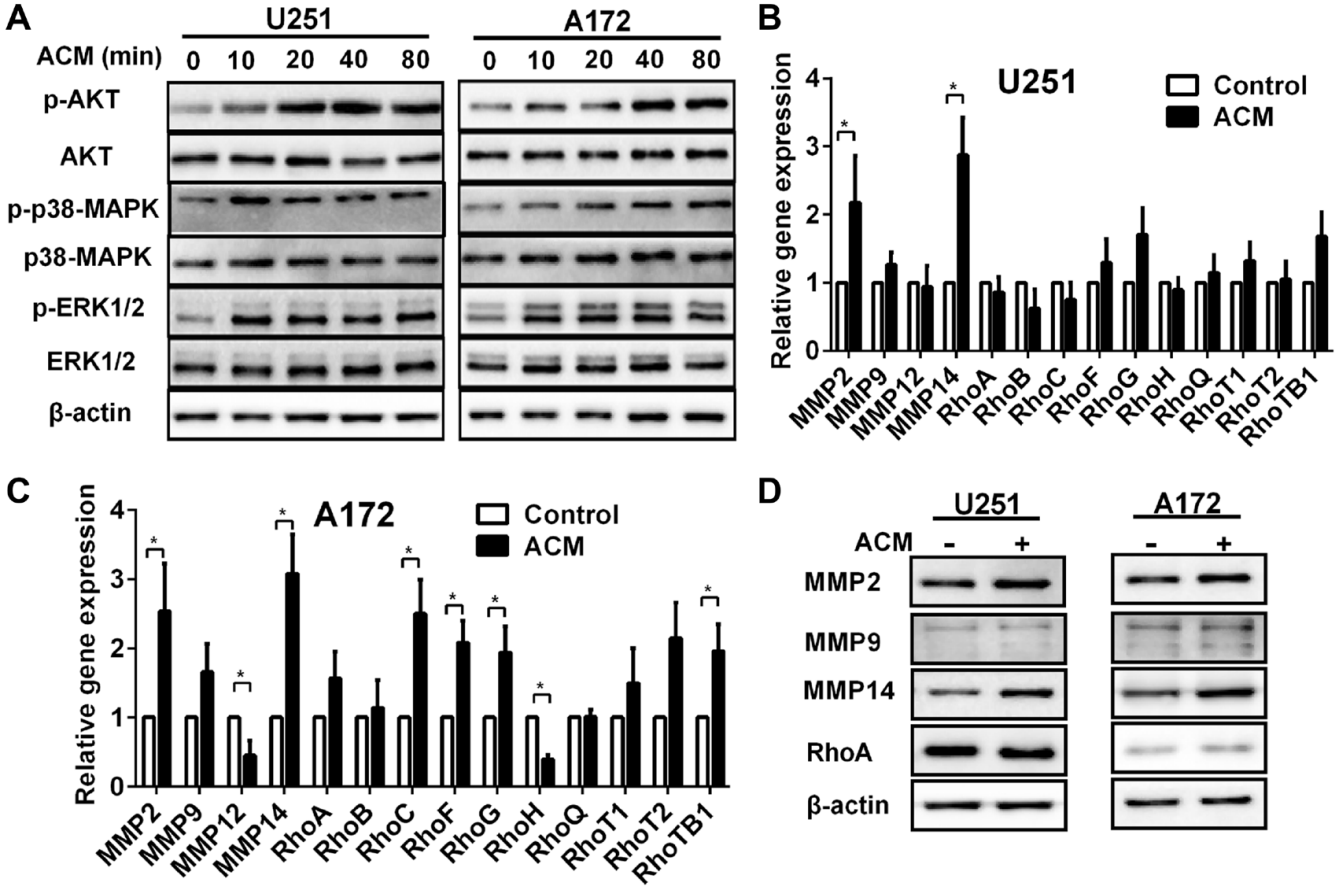
The activated signaling pathways and upregulation of gene and protein associated with invasion induced by astrocytes. (**A**) Western blot analysis of protein lysates prepared from U251 or A172 exposed to astrocytes condition medium (ACM) for the times indicated. (**B**, **C**) Graphic representations of qRT-PCR results for invasion related gene expression changes induced in U251 or A172 in co-culture with astrocytes. Total RNA was extracted from U251 or A172 glioma cells were incubated with ACM for 48 h where DMEM containing 3% FBS was used as the control. ^*^ *p* < 0.05. (**D**) Western blot analysis performed with protein lysates prepared U251 or A172 cells after incubation with ACM for 48 h. Proteins examined are indicated.

**Figure 3:**
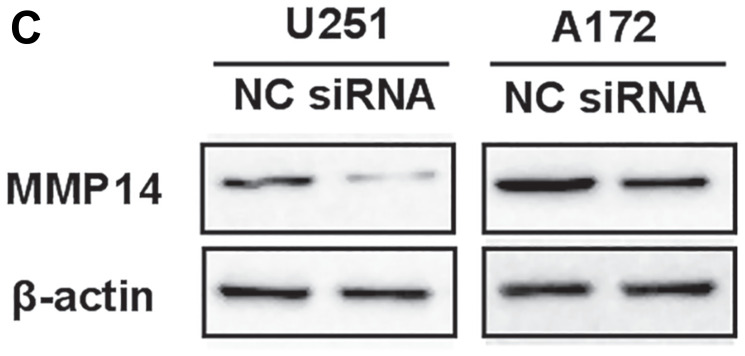
Cytomembrane MMP14 in glioma cell lines is up-regulated by astrocytes in glioma cell lines, and promotes invasion and migration through activation of MMP2 but not cleavage of CD44. (**C**) Western blot analysis for MMP14 48 h after transfection of U251 or A172 cells with siRNA-MMP14 or negative control sequences (NC).

**Figure 4:**
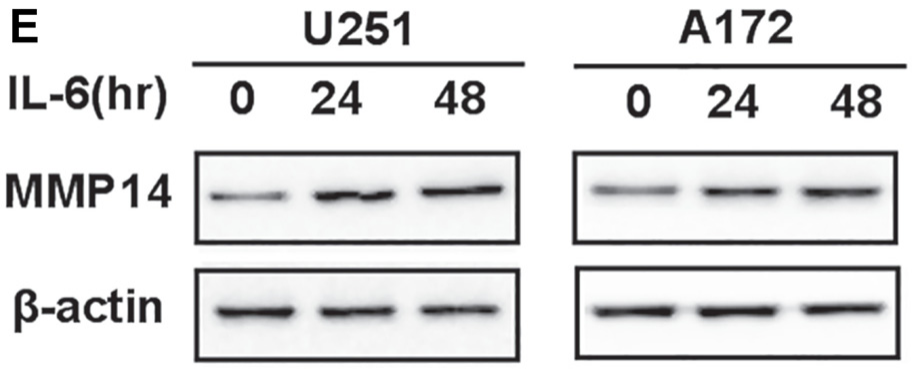
IL-6 secreted by astrocytes induces cytomembrane MMP14 expression on glioma cells. (**E**) Western blot analysis for total MMP14 protein performed with protein lysates prepared from U251 or A172 treated with IL-6 (50 ng/mL) for 48 h.

