# Supplementary material for: Human astrocytes secrete IL-6 to promote glioma migration and invasion through upregulation of cytomembrane MMP14
Source: Oncotarget. 2016 Aug 23;7(38):62425–38. doi: 10.18632/oncotarget.11515 (PMC5308737; doi:10.18632/oncotarget.11515)
Supplement: Supplementary file 1 [file oncotarget-07-62425-s001.pdf]

Human astrocytes secrete IL-6 to promote glioma migration and invasion through upregulation of cytomembrane MMP14

SUPPLEMENTARY FIGURES

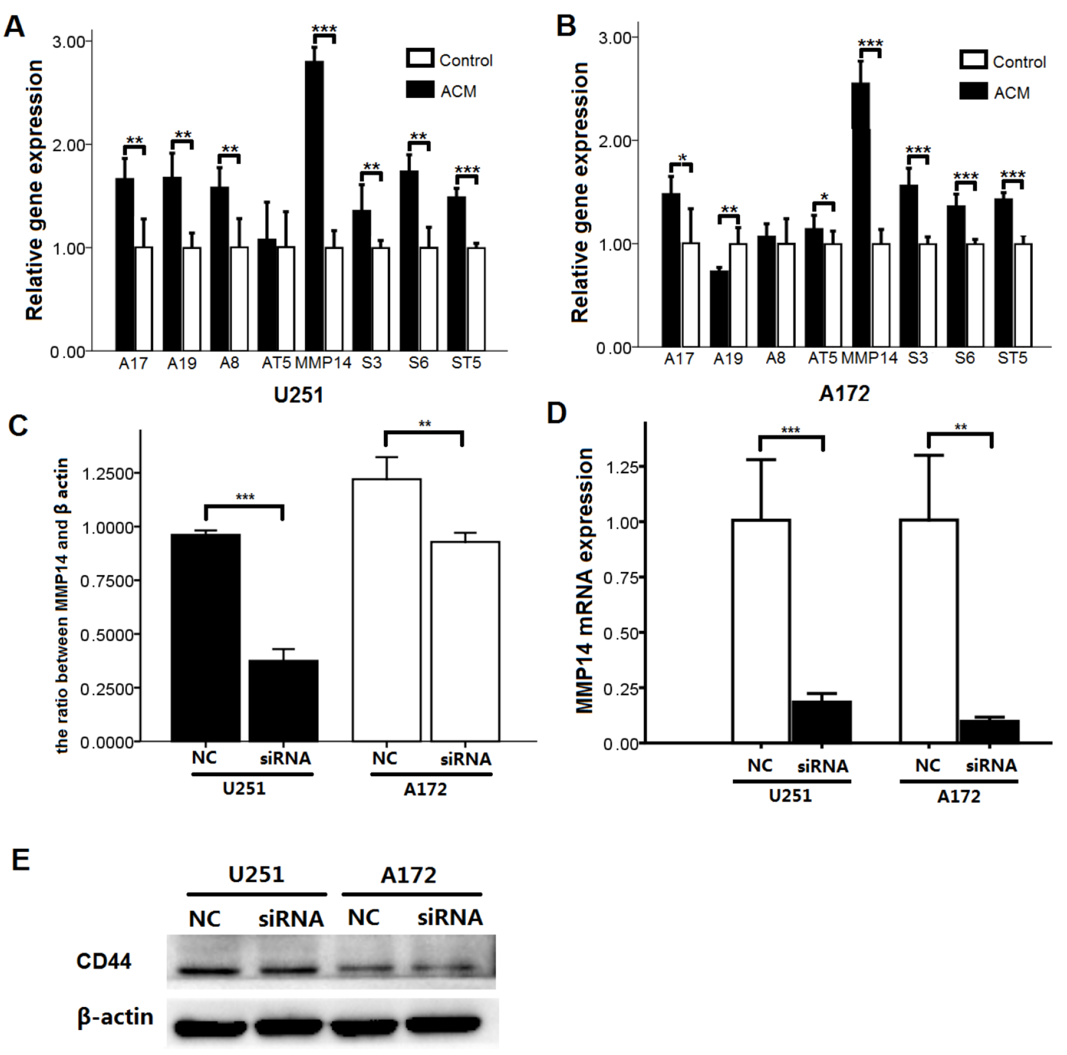

Supplementary Figure S1: qRT-PCR to detect invasion related gene expression changes induced by astrocytes. A. U251 or **B.** A172 glioma cells were incubated with ACM for 48 h and DMEM containing 3% FBS was used as the control. **C.** Western blot to determine protein levels for MMP14 in U251 or A172 cells 48 h after transfection siRNA targeting MMP14 or negative control sequences (NC). **D.** MMP14 mRNA expression in total RNA prepared from U251 and A172 quantitated by qRT-PCR 48 h after transfection with siRNA-MMP14 or negative control sequences (NC).Glioma cells were transfected with siRNA targeting MMP14 (siRNA-MMP14) or negative control sequences (NC). After 48 h, total RNA was isolated and MMP14 mRNA was quantitated with qRT-PCR. **E.** Western blot displaying CD44 protein levels 48 h after transfection with siRNA-MMP14 or negative control sequences (NC). All above data are shown as the mean ± SD in three independent experiments. * $p < 0.05$; ** $p < 0.01$; *** $p < 0.001$.

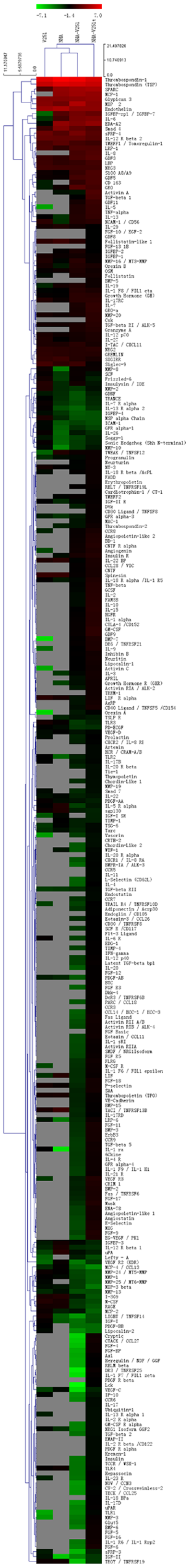

Supplementary Figure S2: Heat Map and Hierarchical Clustering. The heat map diagram shows the result of the two-way hierarchical clustering of antibodies and samples. Each row represents an antibody and each column represents a sample. The antibody clustering tree is shown on the left, and the sample clustering tree appears at the top. The color scale shown at the top illustrates the relative expression level of an antibody in the certain slide: red color represents a high relative expression level; green color represents a low relative expression levels.
